# Global prospective case series of ERCPs using a single-use duodenoscope

**DOI:** 10.1055/a-2131-7180

**Published:** 2023-09-20

**Authors:** Marco J. Bruno, Torsten Beyna, David Carr-Locke, Prabhleen Chahal, Guido Costamagna, Benedict Devereaux, Marc Giovannini, Mahesh K. Goenka, Christopher Khor, James Lau, Gary May, V. Raman Muthusamy, Sandeep Patel, Bret T. Petersen, Douglas K. Pleskow, Isaac Raijman, D. Nageshwar Reddy, Alessandro Repici, Andrew S. Ross, Divyesh V. Sejpal, Stuart Sherman, Uzma D. Siddiqui, Christopher Ziady, Joyce A. Peetermans, Matthew J. Rousseau, Adam Slivka, EXALT Single-use Duodenoscope Study Group

**Affiliations:** 1 Department of Gastroenterology and Hepatology, Erasmus Medical Center, University Medical Center, Rotterdam, The Netherlands; 2 Department of Internal Medicine, Evangelisches Krankenhaus Düsseldorf, Düsseldorf, Germany; 3 Division of Gastroenterology & Hepatology, New York Presbyterian Hospital, Weill Cornell Medicine, New York, New York, USA; 4 Gastroenterology and Hepatology, Digestive Disease and Surgery Institute, Cleveland Clinic, Cleveland, Ohio, USA; 5 Fondazione Policlinico Universitario Agostino Gemelli IRCCS (Università Cattolica del Sacro Cuore), Rome, Italy; 6 Department of Gastroenterology, University of Queensland, Royal Brisbane and Women’s Hospital, Brisbane, Australia; 7 Endoscopy Unit, Institut Paoli-Calmettes, Marseille, France; 8 Department of Gastroenterology, Apollo Multispecialty Hospitals, Kolkata, India; 9 Department of Gastroenterology and Hepatology, Singapore General Hospital, Singapore; 10 Prince of Wales Hospital, Hong Kong, China; 11 The Centre for Therapeutic Endoscopy and Endoscopic Oncology, Division of Gastroenterology, St. Michael’s Hospital, Alberta, Canada; 12 Vatche and Tamar Manoukian Division of Digestive Diseases, David Geffen School of Medicine at UCLA, University of California, Los Angeles, California, USA; 13 Division of Gastroenterology, UT Health San Antonio, San Antonio, Texas, USA; 14 Division of Gastroenterology and Hepatology, Mayo Clinic, Rochester, Minnesota, USA; 15 Center for Advanced Endoscopy, Division of Gastroenterology, Beth Israel Deaconess Medical Center, and Harvard Medical School, Boston, Massachusetts, USA; 16 Texas International Endoscopy Center, Houston, Texas, USA; 17 Asian Institute of Gastroenterology, Hyderabad, India; 18 Endoscopy Unit, Humanitas Clinical and Research Center IRCCS, Rozzano, Milan, Italy; 19 Department of Biomedical Sciences, Humanitas University, Rozzano, Milan, Italy; 20 Department of Gastroenterology, Digestive Disease Institute, Virginia Mason Medical Center, Seattle, Washington; 21 Digestive Disease Institute, Dignity/CommonSpirit Health, Creighton School of Medicine, Phoenix, Arizona, USA; 22 Division of Gastroenterology and Hepatology, Department of Medicine, Indiana University, Indianapolis, Indiana, USA; 23 Center for Endoscopic Research and Therapeutics (CERT), University of Chicago, Chicago, Illinois, USA; 24 Dr. George Mukhari Academic Medical Center, Pretoria, South Africa; 25 Endoscopy Division, Boston Scientific Corporation, Marlborough, Massachusetts, USA; 26 Department of Gastroenterology, Hepatology, and Nutrition, University of Pittsburgh Medical Center, Pittsburgh, Pennsylvania, USA

## Abstract

**Background** The first commercialized single-use duodenoscope was cleared by the US Food and Drug Administration in December 2019. Data regarding endoscopic retrograde cholangiopancreatography (ERCP) using a single-use duodenoscope are needed on a broader range of cases conducted by endoscopists with varying levels of experience in a wide range of geographic areas.

**Methods** 61 endoscopists at 22 academic centers in 11 countries performed ERCP procedures in adult patients aged ≥ 18. Outcomes included ERCP completion for the intended indication, rate of crossover to a reusable endoscope, device performance ratings, and serious adverse events (SAEs).

**Results** Among 551 patients, 236 (42.8 %) were aged > 65, 281 (51.0 %) were men, and 256 (46.5 %) had their procedure as an inpatient. ERCPs included 196 (35.6 %) with American Society for Gastrointestinal Endoscopy complexity of grades 3–4. A total of 529 ERCPs (96.0 %) were completed: 503 (91.3 %) using only the single-use duodenoscope, and 26 (4.7 %) with crossover to a reusable endoscope. There were 22 ERCPs (4.0 %) that were not completed, of which 11 (2.0 %) included a crossover and 11 (2.0 %) were aborted cases (no crossover). Median ERCP completion time was 24.0 minutes. Median overall satisfaction with the single-use duodenoscope was 8.0 (scale of 1 to 10 [best]). SAEs were reported in 43 patients (7.8 %), including 17 (3.1 %) who developed post-ERCP pancreatitis.

**Conclusions** In academic medical centers over a wide geographic distribution, endoscopists with varying levels of experience using the first marketed single-use duodenoscope had good ERCP procedural success and reported high performance ratings for this device.

## Graphical abstract

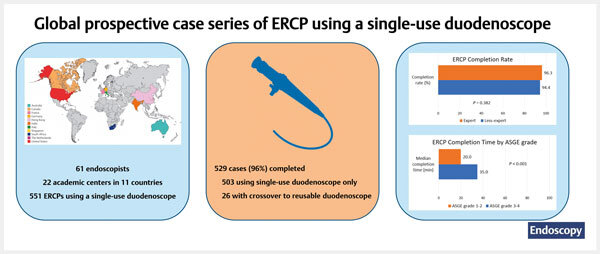

## Introduction

Post-endoscopic retrograde cholangiopancreatography (ERCP) infections are estimated to occur in approximately 5 % of patients 1 2 , commonly due to enteric organisms 3 . Of greatest concern are infections from multidrug-resistant organisms (MDRO), which account for a disproportionately higher morbidity and mortality compared with infections with antibiotic-susceptible organisms 4 . These can be endogenous infections due to translocation of organisms from the gastrointestinal (GI) tract of the patient into the pancreaticobiliary ducts by the duodenoscope or endoscopic devices, or can be exogenous, due to MDROs that were colonizing the ducts of another patient being harbored inside a reusable duodenoscope. A 2022 systematic review estimated the minimum risk of duodenoscope-associated MDRO infection to be at least 0.01 % per ERCP procedure in the Netherlands, which was substantially higher than reported rates in frequently cited older data 5 . The high morbidity and mortality associated with MDRO infections 6 and the delayed clinical detection of some of these infections 7 warrant improved reprocessing practices and the development of a uniform and practical protocol for duodenoscope microbiological sampling that can be applied in general healthcare facilities 8 .

An alternative strategy to address exogenous MDRO infections associated with duodenoscopes is the use of single-use duodenoscopes. Since receiving US Food and Drug Administration (FDA) clearance in 2019, the first single-use duodenoscope has been studied in both US and European settings 9 10 11 and by endoscopists with varying levels of experience 12 . A randomized trial 10 and three case series 9 11 12 reported comparable safety profiles, technical performance, and endoscopist user satisfaction ratings for the first marketed single-use duodenoscope compared with reusable duodenoscopes. A second brand of single-use duodenoscope was cleared in 2020 13 . Additional data are warranted, particularly to document the performance of the single-use duodenoscopes when used by less expert endoscopists, in cases with high complexity, and across a broad geographic range.

To characterize the efficacy and safety of the first marketed single-use duodenoscope further, we conducted a large clinical case series of ERCPs of any complexity in academic medical centers.

## Methods

### Study design

This was a large, multinational case series of the performance of a single-use duodenoscope for ERCP. Institutional Review Board and Ethics Committee approvals for the study were obtained at all study sites. All enrolled patients provided written informed consent for study participation before they contributed data. Single-use duodenoscopes were provided without charge to the investigational sites for use in the study procedures.

Boston Scientific Corporation (Marlborough, Massachusetts, USA) sponsored and funded the study. Sixteen endoscopists participated in published research during development of the single-use duodenoscope 9 12 14 . A statistician (M.J.R.) who is a full-time employee of Boston Scientific Corporation performed the data management and statistical analysis of this study. Review and input to the analysis of the study data were provided by two Boston Scientific employees (J.A.P. and M.J.R.) and by all participating physicians at Investigator Meetings between 2019 and 2022.

### Single-use duodenoscope

The device used in this study was the EXALT Model D single-use duodenoscope (Boston Scientific Corporation), a sterile, single-use duodenoscope designed to function similarly to currently marketed reusable duodenoscopes, and to be discarded after use in a single procedure. The single-use duodenoscope received US FDA clearance in December 2019 and gained a CE mark in January 2020 15 16 .

### Patient population

The study protocol allowed for recruitment of adult patients scheduled for ERCP per the standard of care at up to 40 participating healthcare centers. Eligible patients were recruited for the study on selected weekdays when participating endoscopists were performing ERCPs. Patients were eligible for inclusion if they were aged ≥ 18 years, willing and able to comply with the study procedures and to provide written informed consent to participate in the study, and were scheduled for a clinically indicated ERCP or other duodenoscope-based procedure. Excluded from the study were potentially vulnerable patients including, but not limited to: pregnant women, patients for whom endoscopic techniques were contraindicated, patients enrolled in another investigational study that would directly interfere with the current study, and patients excluded at investigator discretion. Unlike previous studies of the single-use duodenoscope, the current study included patients with “altered pancreaticobiliary anatomy” because this was not a contraindication in the Directions for Use approved in 2020 17 .

### Study procedures

#### Patient assessments

All enrolled participants had a preprocedural study visit for assessment of their demographics and relevant medical history. After the index procedure, participants were evaluated in person or by telephone at 72 hours (−1 day to + 2 days) and again at 30 days (± 3 days) to screen for post-procedure adverse events (AEs) or resolution of any previously reported issues. If the last follow-up visit was not completed, the reason was noted on the study completion form. Participants were considered lost to follow-up if they failed to return for their scheduled follow-up visits and were unable to be contacted by the study site staff after at least three documented attempts.

#### ERCP procedures

Participating endoscopists agreed to use the single-use duodenoscope in place of other brands of duodenoscope(s) used in the endoscopy unit for ERCP. Endoscopists’ level of experience was categorized as “expert” (> 2000 lifetime ERCPs) and “less expert” (≤ 2000 lifetime ERCPs). Start and stop times of the procedure, post-procedure subjective feedback on performance-related attributes, American Society for Gastrointestinal Endoscopy (ASGE) grade for complexity of the ERCP procedure, and all attempted or completed maneuvers during the ERCP were documented. Device deficiencies were recorded and reported, regardless of whether they led to an AE or inability to complete the ERCP. If the necessary maneuvers could not be completed with the single-use device and the endoscopist crossed over to use a reusable duodenoscope, the reason for noncompletion with the single-use device was recorded, and the ability to complete the ERCP maneuvers with a reusable duodenoscope was documented.

Difficult common bile duct (CBD) cannulation was defined by a modification of the European Society of Gastrointestinal Endoscopy (ESGE) definition 18 as the presence of one or more of the following: more than five contacts with the papilla while attempting to cannulate; more than 5 minutes spent attempting to cannulate following visualization of the papilla; at least one (instead of “more than one”) unintended pancreatic duct cannulation or opacification 18 . To represent all difficult procedures, difficult CBD cannulation was tabulated with failed ERCPs (even if cannulation was achieved).

### Outcomes

The primary end point was the ability to complete the ERCP procedures for the intended indication(s). The secondary end points were: (i) the incidence of crossover from the single-use duodenoscope to a reusable duodenoscope; (ii) comparison of outcomes by ASGE grade, endoscopist level of experience, or prior sphincterotomy; (iii) endoscopist ratings of overall satisfaction with the single-use duodenoscope on a scale of 1 (worst) to 10 (best); (iv) serious AEs (SAEs) related to the device and/or procedure, assessed through to 30 days after the ERCP or other duodenoscope-based procedure.

### Statistical analysis

Descriptive statistics included: frequency statistics for patient and procedural characteristics and procedure completion rates (completion rates reported separately for cases with and without crossover to a reusable duodenoscope); median and range for overall satisfaction and procedure duration; and mean (SD) and range for age and mean number of cannulation attempts. Endoscopist experience and ASGE complexity grades were assessed for completion time and performance ratings using Wilcoxon rank-sum tests, and for number of cannulation attempts using a negative binomial model. Fisher’s exact test was used to compare the occurrence of crossover to a reusable duodenoscope by level of experience or by ASGE case complexity level. For binary data, 95 %CIs were calculated using the Clopper–Pearson exact methods. Statistical analyses were performed using SAS 9.4 software (SAS Institute Inc., Cary, North Carolina, USA).

## Results

### Enrollment and patient characteristics

**Fig. 1:**
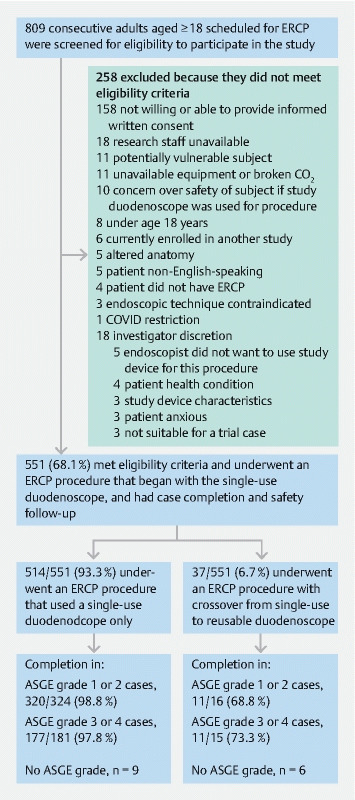
Patient flow through the study.

Of 809 patients who were screened, 551 (68.1 %) were enrolled after providing written informed consent to participate in the study. Reasons for nonparticipation are summarized in Fig. 1 .

Of the 551 patients (mean age 59.9 years) who had a procedure, 281 (51.0 %) were male and 332 (60.3 %) had a prior ERCP ( Table 1 ), with 243 patients (44.1 %) having had a prior biliary and/or pancreatic sphincterotomy. The most common prior GI disorders in patients scheduled for ERCP or other duodenoscope-based procedure were: current bile duct stones/gallstones (n = 168; 30.5 %), biliary or pancreatic duct stricture (n = 124; 22.5 %), current immunosuppression for any cause (n = 71; 12.9 %), chronic pancreatitis (n = 68; 12.3 %), pancreatic tumor (n = 63; 11.4 %), abnormal imaging finding (n = 60; 10.9 %), current cholangitis (n = 53; 9.6 %), or cholangiocarcinoma (n = 46; 8.3 %). Two patients (0.4 %) had a known history of MDRO colonization.

**Table 1:** Characteristics of the 551 patients who were entered into the study and underwent an ERCP procedure that began with a single-use duodenoscope.

Characteristic	n (%), unless otherwise specified
Age, mean (SD) [range], years	59.9 (15.9) [18.0−94.0]
Older than 65 years	236 (42.8)
Sex, male	281 (51.0)
American Society of Anesthesiology physical status	
I	66 (12.0)
II	198 (35.9)
III	250 (45.4)
IV	32 (5.8)
V	0 (0.0)
Not assessed	5 (0.9)
Relevant gastrointestinal medical history	
Bile duct stones/gallstones(current)	168 (30.5)
Documented biliary or pancreatic stricture, unresolved	124 (22.5)
Current immunosuppression (any cause)	71 (12.9)
Pharmacologically induced	64 (11.6)
Post-transplant	30 (5.4)
Post-chemotherapy for cancer other than bile duct	19 (3.4)
Post-chemotherapy for bile duct cancer	12 (2.2)
Disease induced	11 (2.0)
In preparation for bone marrow or other transplantation	1 (0.2)
Other	5 (0.9)
Chronic pancreatitis	68 (12.3)
Pancreatic tumor	63 (11.4)
Abnormal image finding	60 (10.9)
Cholangitis (current)	53 (9.6)
Cholangiocarcinoma	46 (8.3)
Primary sclerosing cholangitis	24 (4.4)
Other gastrointestinal cancer	22 (4.0)
Pancreatic stones (current)	22 (4.0)
Recurrent cholangitis	21 (3.8)
Hepatic tumor	18 (3.3)
Hepatitis	11 (2.0)
Pancreatic pseudocyst	9 (1.6)
Known history of MDRO colonization	2 (0.4)
Known current MDRO colonization	2 (0.4)
Current documented bacterial infection, other than cholangitis	8 (1.5)
Other	146 (26.5)

ERCP, endoscopic retrograde cholangiopancreatography; MDRO, multidrug-resistant organism.

Of the 332 patients for whom the referral source was documented, 102 (30.7 %) presented directly to the participating study site, 154 (46.4 %) were referred from an academic medical center, and 76 (22.9 %) from a community medical center.

### General ERCP characteristics

A total of 61 endoscopists (46 expert, 15 less expert) at 22 academic centers in 11 countries (1–6 endoscopists per center) performed 551 procedures: 25 cases at 21 of the study sites and 26 cases in the other. The median number of procedures performed by each endoscopist was seven (range 1–25). All procedures were performed in endoscopy suites, almost all (99.6 %; 549/551) during normal working hours, and most with endoscopy processing staff present (79.1 %; 436 /551) ( Table 2 ). Prophylactic antibiotics were used in 272 (49.4 %) patients, and prophylactic nonsteroidal anti-inflammatory drugs were used in 315 (57.2 %). The ERCPs included all ASGE grades of complexity: grade 1 (least complex; 10.2 %; 56/551), grade 2 (51.5 %; 284/551), grade 3 (28.5 %; 157/551), and grade 4 (most complex; 7.1 %, 39/551). There were also 15 cases (2.9 %) that were duodenoscope-based procedures not included in the ASGE grading system. The median (range) procedure completion time was 24.0 (2.0–157.0) minutes, including times of ≤ 20 minutes in 240/534 cases (44.9 %) to ≥ 60 minutes in 39/534 (7.5 %) cases.

**Table 2:** Characteristics of the 551 endoscopic procedures carried out.

Characteristic	n (%), unless otherwise specified
Inpatient procedure	256 (46.5)
ERCP procedure	545 (98.9)
Prophylactic NSAID used	315 (57.2)
Prophylactic antibiotics used	272 (49.4)
Patient intubated	253 (45.9)
Medications for anesthesia	
Propofol	477 (86.6)
Opioid	178 (32.3)
Neuromuscular blocker	137 (24.9)
Inhalation agent	74 (13.4)
Etomidate	13 (2.4)
Other	163 (29.6)
Medications for maintenance of anesthesia	
Propofol	391 (71.0)
Opioid	153 (27.8)
Sevoflurane	124 (22.5)
Benzodiazepine	94 (17.1)
Neuromuscular blocker	54 (9.8)
Other	74 (13.4)
Procedure time with single-use duodenoscope, median (range) [n], minutes 1	22.0 (0.0−157.0) [433]
Reusable scope used	37 (6.7)
Procedure time with reusable duodenoscope, median (range) [n], minutes	22.0 (6.0−75.0) [37]
Patient position	
Prone	379 (68.8)
Supine	133 (24.1)
Left lateral decubitus	52 (9.4)
Location of procedure	
Endoscopy suite	551 (100.0)
ERCP during normal hours	549 (99.6)
Endoscope reprocessing staff present	436 (79.1)
Total procedure time, median (range) [n], minutes 1	24.0 (2.0−157.0) [548]
Difficult cannulation or failed ERCP 2	150 (29.2)
Difficult cannulation without failed ERCP 2	140 (27.2)
Failed ERCP without difficult cannulation	4(0.8) (514)
Failed ERCP with difficult cannulation	6(1.2) (514)
All intended maneuvers completed	524 (95.1)
ERCP completion	
Completed, no crossover	503 (91.3)
Completed with crossover	26 (4.7)
Not completed, without crossover	11 (2.0)
Not completed, with crossover	11 (2.0)
Pancreatic maneuvers performed	128 (23.2)
Cholangioscopy	24 (4.4)
Pancreatoscopy	9 (1.6)
ASGE complexity grade 3	
Grade 1	56 (10.2)
Grade 2	284 (51.5)
Grade 3	157 (28.5)
Grade 4	39 (7.1)
Procedures not included in ASGE grading system	15 (2.7)

ERCP, endoscopic retrograde cholangiopancreatography; NSAID, nonsteroidal anti-inflammatory drug; ASGE, American Society for Gastrointestinal Endoscopy.

1 One case was aborted (because patient had food in the stomach) so the procedure time was 0 and was not included in the “total procedure time” calculation.

2 Denominator = 514 procedures.

3 ASGE grade for complexity of ERCPs: grade 1, deep cannulation of duct of interest, main papilla, sampling; grade 2, biliary stone extraction < 10 mm, treat biliary leaks, treat extrahepatic benign and malignant strictures, placed prophylactic stents; grade 3, biliary stone extraction ≥ 10 mm, minor papilla cannulation in divisum and therapy, removal of internally migrated biliary stents, intraductal imaging/biopsy/fine needle aspiration, management of acute or recurrent pancreatitis, treat pancreatic strictures, remove pancreatic stones mobile and < 5 mm, treat hilar tumors, treat benign biliary strictures hilum and above, manage suspected sphincter of Oddi dysfunction (with or without manometry); grade 4, remove internally migrated pancreatic stents, intraductal image-guided therapy (e. g. photodynamic therapy, electrohydraulic lithotripsy), pancreatic stones impacted and/or ≥ 5 mm, intrahepatic stones, pseudocyst drainage/necrosectomy, ampullectomy, ERCP after Whipple or Roux-en-Y bariatric surgery.

Investigators reported that after the study procedures, most of the single-use duodenoscopes were discarded in standard medical waste (35.4 %; 195 /551), or medical grade recycling (29.9 %; 165/551), regulated medical waste (24.9 %; 137/551), a sharps disposal container (4.9 %; 27/551), or other (4.9 %; 27/551). A high proportion of patients completed the 72-hour (92.9 %; 512/551) and 30-day (89.8 %; 495/551) study follow-up.

### Cannulation details

Difficult CBD cannulation was reported for 150 /514 cases (29.2 %) using the single-use duodenoscope (10 of which were failed ERCPs), and in 15 cases in which a reusable duodenoscope was used after crossover.

Advanced cannulation techniques performed included: precut (access) papillotomy (n = 42; 7.6 %), double wire (n = 31; 5.6 %), pancreatic duct plastic stent (n = 24; 4.4 %), rendezvous with EUS (n = 3; one also had precut but ERCP still failed and went to rendezvous), cholangioscopy (n = 2), papillectomy (n = 1), transpancreatic sphincterotomy (n = 1), clip used to bring hidden papilla out from fold (n = 1), and accessory papilla cannulation (n = 1).

### Ability to complete ERCP for intended indication

Among the 551 study cases, 529 (96.0 %, 95 %CI 94.0 %–97.5 %) were completed for the intended indication, including 503 (91.3 %, 95 %CI 88.6 %–93.5 %) using only the single-use duodenoscope, and 26 (4.7 %, 95 %CI 3.1 %–6.8 %) including crossover from the single-use to a reusable duodenoscope. Of the 22 cases that were not completed, 11 included crossover to a reusable duodenoscope ( Table 2 ).

A total of 514 patients (93.3 %) underwent an ERCP procedure that used the single-use duodenoscope only ( Fig. 1 ). Of these, 324 were ASGE grade 1 or 2 cases (98.8 % completed), 181 were ASGE grade 3 or 4 cases (97.8 % completed), and nine did not have an ASGE grade (66.7 % completed). The remaining 37 patients (6.7 %) underwent an ERCP procedure with crossover from single-use to reusable duodenoscope. Of these, 16 were ASGE grade 1 or 2 cases (68.8 % completed), 15 were ASGE grade 3 or 4 cases (73.3 % completed), and six did not have an ASGE grade (66.7 % completed).

For 524 patients (95.1 %), all of the intended maneuvers were completed. Over 20 different types of maneuvers were performed using the single-use duodenoscope, including sphincterotomy, papillectomy/ampullectomy, cannulation, mechanical lithotripsy, clearance of bile duct or pancreatic duct stones, biliary or pancreatic stent placement or removal, balloon dilation, cholangioscopy (n = 24), pancreatoscopy (n = 9), cytology brushing, biopsy, and others. Pancreatic maneuvers were performed in 128 patients (23.2 %).

### Comparison of outcomes by ASGE grade of complexity

The median time of completion was significantly shorter (20.0 vs. 35.0 minutes; *P* < 0.001), and the mean number of cannulation attempts was significantly higher (3.0 vs. 2.4; *P* = 0.01) for ASGE grade 1 or 2 procedures compared with grade 3 or 4 procedures, while ERCP completion (97.4 % [331/340] vs. 95.9 % [188/196]; *P* = 0.44) and crossover rate (4.7 % [16/340] vs. 7.7 % [15/196]; *P* = 0.18) were similar ( Table 3 ).

**Table 3:** Comparison of outcomes by ASGE grade of complexity (n = 536) 1 .

Outcome	Grade 1 or 2 (n = 340)	Grade 3 or 4 (n = 196)	*P* value
ERCP completed, n (%)	331 (97.4)	188 (95.9)	0.44
Crossover rate, n (%) 2	16 (4.7)	15 (7.7)	0.18
Overall satisfaction, median (range) [n]	8.0 (2.0−10.0) [340]	8.0 (1.0−10.0) [195]	0.68
Serious adverse event rate, n (%)	47 (13.8)	36 (18.4)	0.17
Procedural completion time, median (range) [n], minutes	20.0 (2.0−132.0) [338]	35.0 (2.0−157.0) [196]	< 0.001
Number of cannulation attempts, mean (SD) (range) [n]	3.0 (4.3) (1.0−28.0) [323]	2.4 (2.8) (1.0−20.0) [179]	0.01

ASGE, American Society for Gastrointestinal Endoscopy; ERCP, endoscopic retrograde cholangiopancreatography.

1 15 cases (2.9 %) were duodenoscope-based procedures not included in the ASGE grading system.

2 Excludes one crossover case that did not have an ASGE grade.

The median rating of overall satisfaction with the performance of the single-use duodenoscope was 8.0 for cases with both ASGE grades 1 or 2 and ASGE grades 3 or 4 ( *P* = 0.68).

### Comparison of outcomes for expert versus less expert endoscopists

ERCPs performed by expert and less expert endoscopists were similar with respect to completion rate (96.3 % [444/461] vs. 94.4 % [85/90], respectively; *P* = 0.38), median procedural completion time (23.0 vs. 27.0 minutes; *P* = 0.15), mean number of cannulation attempts (2.7 vs. 3.1; *P* = 0.24), crossover rate (6.5 % vs. 7.8 %; *P* = 0.65), and proportion of cases with high complexity (36.4 % [168/461] vs. 31.1 % [28/90]) ( Table 4 ). On a scale of 1 (worst) to 10 (best), the median rating of overall satisfaction with the performance of the single-use duodenoscope was 8.0 for expert endoscopists and 7.0 for less expert endoscopists ( *P* < 0.001).

**Table 4:** Comparison of outcomes by endoscopist level of experience.

Outcome	Expert (n = 461)	Less expert (n = 90)	*P* value
ERCP completed, n (%)	444 (96.3)	85 (94.4)	0.38
Crossover rate, n (%)	30 (6.5)	7 (7.8)	0.65
Median overall satisfaction (range)	8.0 (0.0−10.0)	7.0 (2.0−10.0)	< 0.001
Serious adverse event rate, n (%)	77 (16.7)	8 (8.9)	0.08
Procedural completion time, median (range) [n]	23.0 (2.0−157.0) [458]	27.0 (3.0−100.0)	0.15
Number of cannulation attempts, mean (SD) (range) [n]	2.7 (3.7) (1.0−25.0) [428]	3.1 (4.3) (1.0−28.0) [76]	0.24

ERCP, endoscopic retrograde cholangiopancreatography.

### Comparison of outcomes by prior sphincterotomy

On comparison with cases with at least one prior sphincterotomy, cases with no prior sphincterotomy had a higher median total procedural time (25.0 vs. 21.0 minutes; *P* = 0.01), higher mean (SD) number of cannulation attempts (3.6 [4.7] vs. 1.6 [1.9]; *P* < 0.001), and a lower likelihood of having high complexity ( *P* = 0.001). The baseline sphincterotomy status did not correlate with the crossover rate ( *P* = 0.09), ERCP completion rate ( *P* = 0.52), or overall satisfaction with the single-use duodenoscope ( *P* = 0.36).

### Related serious adverse events

Of the 551 enrolled patients, 43 (7.8 %, 95 %CI 5.7 %–10.4 %) experienced at least one SAE related to the device or procedure within a median (range) of 1 (0–28) days post-procedure ( Table 5 ). There were 18 patients (3.3 %, 95 %CI 2.0 %–5.1 %) who developed pancreatitis (mild or moderate [n = 16], severe [n = 2]; post-ERCP [n = 17] reported 0–4 days after the ERCP, acute pancreatitis [n = 1]), six (1.1 %) who experienced pain, five (0.9 %) who had a GI bleed, three (0.5 %) who developed sepsis, two each (0.4 %) who developed cholangitis, cholecystitis, fever, or bacteremia, or had a perforation (0.4 %, 95 %CI 0.04 %–1.3 %; one gastric, one associated with sphincteroplasty during ERCP). The following additional SAEs occurred in one patient each: pneumoretroperitoneum, pneumonia, reintervention for biliary stones, exacerbation of COPD. SAE rates were similar for expert and less expert endoscopists (16.7 % [77/461] vs. 8.9 % [8/90], respectively; *P* = 0.08) and ASGE grade 1 or 2 cases vs. ASGE grade 3 or 4 levels of complexity (13.8 % [47/340] vs. 18.4 % [36/196], respectively; *P* = 0.17).

**Table 5:** Serious adverse events that occurred by 30 days after ERCP (n = 551 total).

	Events, n 1	Patients, n (%)	95 %CI
**Any serious adverse event**	**46**	**43 (7.8)**	**5.7 %−10.4 %**
Pancreatitis	18	18 (3.3)	1.9 %−5.1 %
PEP	17	17 (3.1)	1.8 %−4.9 %
Fatal acute pancreatitis	1	1 (0.2)	0.0 %−1.0 %
Pain	6	6 (1.1)	0.4 %−2.4 %
Gastrointestinal bleeding (fatal in one case)	5	5 (0.9)	0.3 %−2.1 %
Sepsis	3	3 (0.5)	0.1 %−1.6 %
Cholangitis	2	2 (0.4)	0.0 %−1.3 %
Cholecystitis	2	2 (0.4)	0.0 %−1.3 %
Fever	2	2 (0.4)	0.0 %−1.3 %
Bacteremia	2	2 (0.4)	0.0 %−1.3 %
Perforation (fatal in one case)	2	2 (0.4)	0.0 %−1.3 %
Pneumoretroperitoneum	1	1 (0.2)	0.0 %−1.0 %
Reintervention with ERCP under general anesthesia for biliary stone removal	1	1 (0.2)	0.0 %−1.0 %
Post-ERCP pneumonia	1	1 (0.2)	0.0 %−1.0 %
Exacerbation of COPD	1	1 (0.2)	0.0 %−1.0 %

ERCP, endoscopic retrograde cholangiopancreatography; PEP, post-ERCP pancreatitis; COPD, chronic obstructive pulmonary disease.

1 Each patient had one or more of the listed serious adverse events; rows are not mutually exclusive.

The overall rate of related SAEs that were fatal in our study was 0.5 % (3/551; 95 %CI 0.11 %–1.6 %). These included one case of acute pancreatitis occurring 16 days after ERCP that was fatal on day 70, one fatal hemorrhage from the papilla after balloon dilation, and one fatal perforation associated with sphincteroplasty.

## Discussion

In this largest global study of a single-use duodenoscope, endoscopists with varying levels of experience had high procedural success in ERCPs, one-third of which were high complexity cases. The median performance ratings were high for this device. SAEs were in the expected range, based on the published safety data for reusable duodenoscopes. Consistent with two previously published clinical studies 9 12 , the current study supports the safe and effective performance of the first single-use duodenoscope among endoscopists with varying levels of experience in multiple countries.

In an effort to eliminate the potential for contamination or infection associated with ineffective reprocessing of reusable duodenoscopes, the FDA have cleared duodenoscopes with disposable components 19 20 and two fully disposable duodenoscopes 13 15 . Single-use duodenoscopes may be especially useful in certain challenging logistical settings, such as during evening and weekend hours, and in various emergent situations, including when procedures are done outside of the endoscopy department 21 . The disadvantages of single-use duodenoscopes include their current high cost compared with duodenoscopes that are reusable 22 or partially disposable (with disposable endpieces 23 24 ), and their potential adverse impacts on the environment, while recycling programs currently are not yet widely available 25 . Although a 2023 RCT found that duodenoscopes with a disposable elevator cap exhibited reduced contamination following high level disinfection compared with standard scope designs 23 , the status of disposable endoscopic endpieces is uncertain after the FDA issued a warning letter to a duodenoscope manufacturer regarding its slow response to complaints about the distal end cover “dropping out” or cracking 26 .

Ecological sustainability is an important issue for both single-use and reusable devices. For example, a 2023 publication focused on manufacturing-associated carbon dioxide emissions reported that performing ERCP with single-use duodenoscopes releases a carbon dioxide equivalent that is 24–47 times greater than with a reusable duodenoscope or a reusable duodenoscope with disposable endcaps 27 . This report made some assumptions that were not accurate for the EXALT duodenoscope and did not include selective use or recycling in the analysis. Furthermore, the report did not mention ethylene oxide, which is used for approximately 50 % of sterile devices sold in the USA and is regulated by the US Environmental Protection Agency owing to its carcinogenicity 28 . Improvements in recycling efforts, and control and mitigation of the chemicals used in reprocessing are necessary to optimize patient and employee safety as single-use and reusable duodenoscopes are both used in clinical practice.

The results from the current study are consistent with those from previous studies of the single-use duodenoscope 9 10 11 12 , but from a much larger study population. The device was successfully used for procedures of all ASGE grades of complexity, with a low (6.7 %) incidence of crossover to a reusable duodenoscope and high (96 %) completion rate by both expert and less expert operators. Study practices met the published ERCP quality indicators 29 30 31 for most items that had study documentation. Consistent with the ESGE quality indicators for ERCP 30 , the rate of post-ERCP pancreatitis was < 10 % in our study. Although the precise role and place of single-use devices is the subject of ongoing discussion, the current study provides added evidence that there are no arguments against their use from the perspective of procedural outcome and success, which is a prerequisite for considering their use in clinical practice.

All ERCP-associated SAEs are concerning and continual efforts must be made to minimize them. We compared the safety results in the current study with the published safety data for procedures performed with reusable duodenoscopes. The largest analysis of ERCP safety available is from Andriulli et al. 32 , who estimated the post-ERCP complication rates among 21 prospective studies published between January 1977 and May 2006. They reported an estimated ERCP-specific mortality rate of 0.33 % (55/16 855 patients; 95 %CI 0.24 %–0.42 %), which is comparable to the rate of fatal related SAEs in our study (0.5 %; 3/551). Andriulli’s estimated rates of perforation (0.60 %, 95 %CI 0.48 %–0.72 %), pancreatitis (585/16 855 patients; 3.47 %, 95 %CI 3.19 %–3.75 %), and infection (1.44 %; 95 %CI 1.26 %–1.62 %) were also similar to the rates in our study (0.4 % [2/551], 3.3 % [18/551], and 1.63 % [9/551; 95 %CI 0.75 %–3.08 %], respectively).

These findings suggest that, when used by a group of endoscopists with a predominantly expert level of experience, the single-use duodenoscope has a comparable safety profile to reusable duodenoscopes. The comparable rate of infection also shows that endogenous infection can still occur with when using a single-use duodenoscope, so the procedure-associated infection rate is not guaranteed to be lower than the rate for reusable duodenoscopes.

Our study has several strengths, limitations, and considerations for study interpretation. This was a multinational observational study including a large number of scheduled ERCP cases with a range of complexity performed by endoscopists at varying levels of experience, including 30-day safety follow-up. Limitations include the lack of randomization and the absence of a control group. Although patients were consecutively screened, the 68 % who were eligible to participate in this study may not be typical of patients at all endoscopy centers. Specific reasons why 10 screened patients were excluded for “concern over safety of subject if study duodenoscope was used for procedure’’ were not documented. Of note, several investigators participated in the development of the single-use duodenoscope 9 14 and have also received research funding from the study sponsor and from manufacturers of reusable duodenoscopes. Finally, this study evaluated the efficacy and safety of a single-use duodenoscope but was not designed to address the associated costs or impact on reprocessing services or the environment.

In conclusion, academic endoscopists used the first marketed single-use duodenoscope to successfully complete a large number of scheduled ERCP procedures with a range of complexity in a diverse patient population. Consistent with past studies, the device showed good performance and an SAE rate comparable with the published estimates for reusable duodenoscopes.

## Data sharing

The data, analytic methods, and study materials for this study may be made available to other researchers in accordance with the Boston Scientific Data Sharing Policy ( https://www.bostonscientific.com/en-US/data-sharing-requests.html
